# Factors Associated with the Prevalence of Circulating Antigens to Porcine Cysticercosis in Three Villages of Burkina Faso

**DOI:** 10.1371/journal.pntd.0000927

**Published:** 2011-01-04

**Authors:** Rasmané Ganaba, Nicolas Praet, Hélène Carabin, Athanase Millogo, Zékiba Tarnagda, Pierre Dorny, Sennen Hounton, Adama Sow, Pascal Nitiéma, Linda D. Cowan

**Affiliations:** 1 Agence de formation, de recherche et d'expertise et en Santé pour l'Afrique (AFRICSanté), Bobo-Dioulasso, Burkina Faso; 2 Institute of Tropical Medicine, Antwerp, Belgium; 3 Department of Biostatistics and Epidemiology, College of Public Health, University of Oklahoma Health Sciences Center, Oklahoma City, Oklahoma, United States of America; 4 Centre Hospitaltier Universitaire Souro Sanou, Bobo-Dioulasso, Burkina Faso; 5 Institut de Recherche en Sciences de la Santé, Bobo-Dioulasso, Burkina Faso; 6 Sexual and Reproductive Health Branch, Technical Division, United Nations Population Fund (UNFPA), New York, New York, United States of America; 7 Pan African Tsetse and Trypanosomiasis Eradication Campaign (PATTEC), Bobo-Dioulasso, Burkina Faso; Universidad Nacional Autónoma de México, Mexico

## Abstract

**Background:**

Little is known about porcine cysticercosis in Burkina Faso. We conducted a pilot study to estimate the prevalence of antigens of *Taenia solium* cysticercosis and to identify associated factors in pigs of three villages in Burkina Faso, selected to represent different pig management practices: one village where pigs are allowed to roam freely (Batondo), one village where pigs are penned part of the time (Pabré) and one village with limited pig farming (Nyonyogo).

**Methods/Principal Findings:**

A clustered random sampling design was used. Data on socio-demographic characteristics (source of drinking water, presence of latrines in the household, type and number of breeding animals) and pig management practices were collected using a standardized questionnaire. Blood samples were collected from one pig per household to determine the presence of antigens of the larval stages of *T. solium* by the B158/B60 Ag-ELISA. The associations between seropositivity and socio-demographic and pig management practices were estimated using logistic regression. Proportions of 32.5% (95% CI 25.4–40.3), 39.6% (31.9–47.8), and 0% of pigs, were found positive for the presence of circulating antigens of *T. solium* in Batondo, Pabré, and Nyonyogo, respectively. The results of the logistic regression analyses suggested that people acquire knowledge on porcine cysticercosis following the contamination of their animals. The presence of antigens in the pigs' sera was not associated with the absence of latrines in the household, the source of drinking water or the status of infection in humans but was associated with pig rearing practices during the rainy season.

**Conclusions/Significance:**

The results suggest that education of pig farmers is urgently needed to reduce the prevalence of this infection.

## Author Summary

*Taenia solium* cysticercosis is a neglected tropical infection transmitted between humans and pigs. This infection is particularly common in areas where sanitation, hygiene and pig management practices are poor, and can sometimes lead to epilepsy in humans. There is very little information about the importance of this infection in Burkina Faso, even though pork meat is widely consumed in many villages. We conducted a pilot study in three villages: two villages where pig rearing and pork consumption are common (Batondo and Pabré) but with different pig management practices, and one village with limited pig farming and pork consumption (Nyonyogo). Blood tests were done on pigs and information on pig raising was collected from farmers. Our study demonstrated that at least one third of pigs are infected with cysticercosis in villages where they are raised, and, particularly when pigs are left to roam some or all of the time. It also demonstrated that farmers may not be aware of this disease until one of their animals is found to be infected. Thus, the study concluded that there is an urgent need for improving education in order to control this tropical disease.

## Introduction

**Figure 1:**
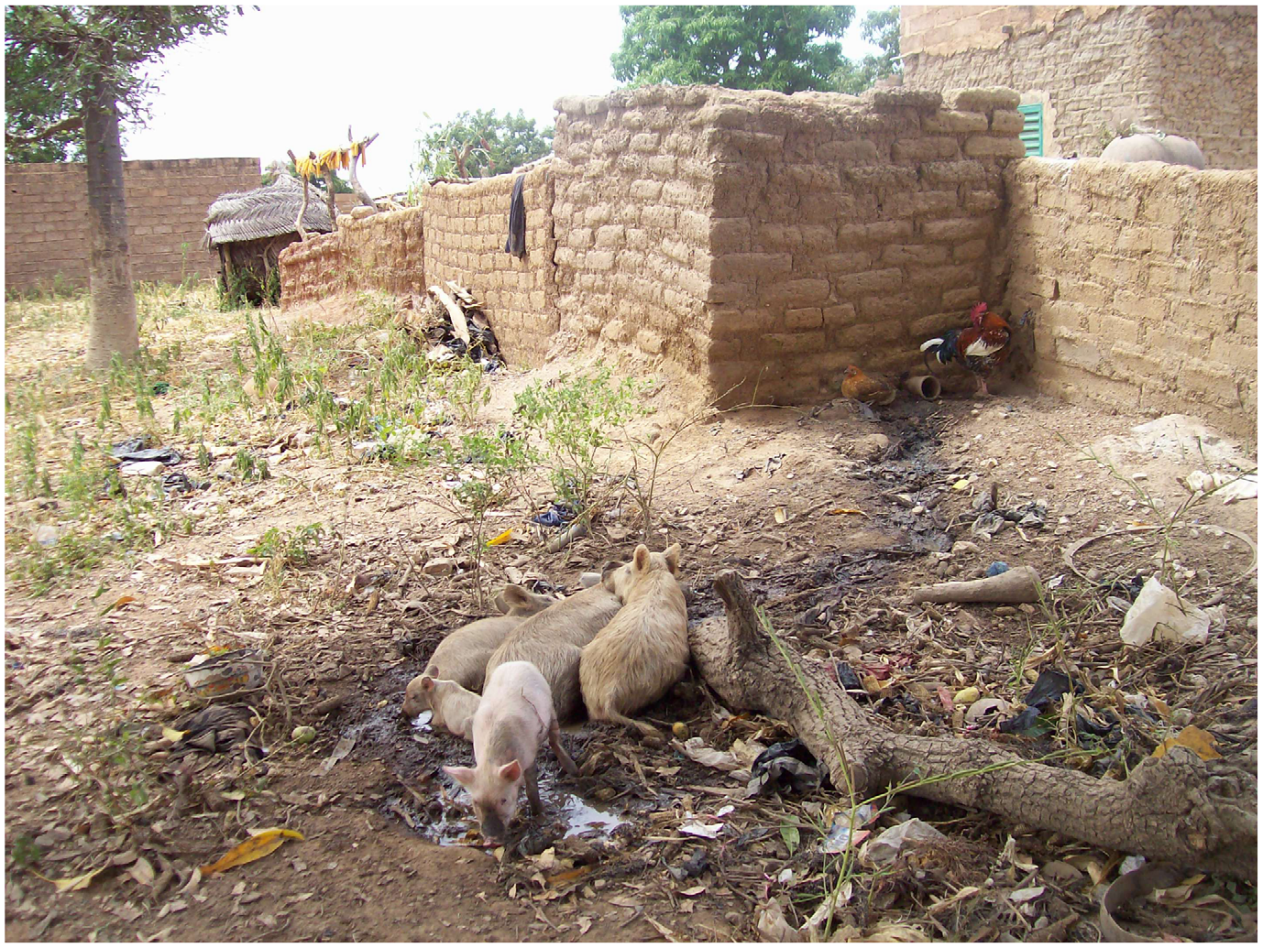
Pigs kept roamed laying in a puddle of water from toilet behind a concession.

Burkina Faso is one of the poorest countries in the world ranking 177^th^ out of 182 according to the Human Development Index (HDI) [1]. Its economy relies predominantly on agriculture (40% of GDP), with cotton production being the most important, followed by livestock production, accounting for 12% of the GDP [2]. The main livestock species are cattle, small ruminants, poultry and pigs. The relative importance of each species varies across the thirteen regions of the country. The country's pig population is estimated at 2 million, and is more concentrated in the regions of Centre West, South West, and Boucle du Mouhoun (with 45% of the pig population) [3]. Pigs are kept mainly in a traditional way, with most being either tethered or allowed to roam freely for some time during the year (Figure 1).

Porcine cysticercosis is a parasitic zoonosis caused by the larval stage of *Taenia solium*. While pigs are the intermediate host, man is the only natural definitive host. Human tapeworm carriers shed thousands of eggs daily through their feces. Pigs usually get infected by eating infected human feces or by consuming feed or water contaminated with human feces. Humans can also become accidental intermediate hosts upon ingestion of *T. solium* eggs. In both humans and pigs, the larval stage of *T. solium* can establish in the muscles and/or in the brain, the latter resulting in neurocysticercosis (NCC). Human NCC may lead to acute seizures, epilepsy and other neurological manifestations [4]. Ingestion of larvae (cysticerci) present in raw or under-cooked pork may result in human tapeworm infection. Porcine cysticercosis is common in developing countries where pigs are raised. Porcine cysticercosis is associated with poor sanitation and hygiene (absence of latrines, defecation in pigpens, poor handwashing practices), poor methods of pig husbandry (free-roaming), lack of meat inspection, and poor knowledge of the disease [5]–[11], all of which are associated with poverty. In order to avoid economic losses due to condemnation of infected pig carcasses in places where meat inspection is performed [10], farmers may sell contaminated pigs, either alive or for clandestine slaughtering.

Information on porcine cysticercosis in Burkina Faso is very limited. Coulibaly and Yameogo (2000) [12] reported a prevalence of cysticercosis of 0.6% among pigs inspected at slaughter. Based on data from the main abattoir in Ouagadougou for 2005 to 2007, of 10,505, 12,651, and 11,887 pigs slaughtered during each of these three years, respectively, only 8 (0.08%), 13 (0.10%), and 10 (0.08%) were condemned because of cysticercosis; whereas 472, 186, and 133 carcasses, respectively, were reportedly condemned because of illegal slaughtering. These data clearly show that inspection of pork is very poor in Burkina, and reliable prevalence of porcine cysticercosis is lacking in the country. The aims of this study were therefore: 1) to estimate the prevalence of *T. solium* cysticercosis among pigs in three villages with distinct pigs raising techniques, and 2) to measure the association between potential risk factors and the prevalence of infection in pigs.

## Methods

We conducted a cross-sectional study in three villages including serological detection of active cysticercosis in pigs and a questionnaire survey for the identification of potential risk factors. In two villages, Batondo and Pabré, located 140 km west and 20 km north of Ouagadougou (the capital of Burkina Faso), respectively, pig breeding and pork consumption are very common. In a third village, Nyonyogo, located 30 km north of Ouagadougou, both pig breeding and pork consumption are rare. The sampling of pigs took place between 26 May and 29 October 2007, which corresponds to the rainy season and the very start of the dry season. The sampling was implemented by a research team composed of one physician, one veterinarian, two interviewers, and one translator (for Batondo only).

### Sampling and measurement of potential risk factors

The first step in the sampling process was to determine the sampling frame. This was done by using information from the national population census conducted in 2006 during which all villages were divided into enumeration areas (EA), a geographical unit which is intended to include approximately 1,000 persons. In each of the three villages, the sampling process started by identifying the geographic limits of each EA. In each EA, each concession (a grouping of several households, usually members of the same family) was numbered. In Batondo (4 EAs) and Nyonyogo (3 EAs), all the concessions were included because of the small number of concessions present. In Pabré (5 EAs), 50% of all concessions were randomly selected.

In each concession, all the households were invited to participate. The head of each household was asked to list all the members of the household and the mother or the oldest woman (in the case of polygamy) was asked questions regarding selected characteristics of the household, such as the source of drinking water, the presence of latrines, breeding livestock, and cooking pork. The person in charge of pig farming was asked about how pigs were managed and slaughtered. Knowledge of porcine cysticercosis was assessed through questions about seeing lesions in the meat on dead animals or cysts under the tongue of live animals. Before the data collection started, the questionnaires were validated during a pre-test conducted in Tenado, a village located eight km from Batondo. One pig was randomly sampled per household for blood sample collection. Blood samples were also collected in humans (one person randomly selected per household) (see [13] for more details on the human component of the study). All questionnaires are available on request.

Blood samples were left to decant and the sera were transported to the Institut de Recherche en Sciences de la Santé (IRSS) in Bobo-Dioulasso where they were kept at −20°C until analysis.

### Serological test

The serum samples were tested in duplicate with the enzyme-linked immunosorbent assay (ELISA) for the detection of circulating antigens of the metacestode of *T. solium* (Ag-ELISA) [14]. Sera showing a coefficient of variation of more than 50% between the duplicate results were considered as missing values (n = 10). The Ag-ELISA has been reported to have a sensitivity of 76.3% (95%CI: 60.9%–88.6%), 86.7% (95%CI: 62.0%–98.0%) and 85.8% (95%CI: 71.9%–99.7%) and a specificity of 84.1% (95%CI: 74.4%–93.3%), 94.7% (95%CI: 90.0%–99.7%) and 98.9% (95%CI: 97.3%–100%) in pigs in South Africa, Zambia and West Cameroon, respectively [6], [14], [15].

### Statistical analysis

First, the relative frequencies of categorical variables and the mean and extreme values of quantitative variables were calculated. Wealth quintiles for households were derived from a score calculated based on the asset ownership of each household (e.g., source of drinking water, presence of toilet, radio, bicycle, presence, type and number of livestock, types of roof and walls) using factor analysis as described by Gwatkin *et al.* (2000) [16]. Secondly, bivariate analyses were conducted to assess the associations between pigs' serological status and location (village), human socio-demographic and health factors (source of drinking water, presence of latrine, presence of antigens to the larval stages of *T. solium*, wealth quintile), and pig management factors. Finally, a multiple logistic regression was used to estimate the adjusted associations between the factors mentioned above and the presence of antigens to *T. solium* in pigs.

Data from Nyonyogo were excluded from the logistic regression because of the small number of pigs in the village (only 6 pigs were sampled there). All the analyses were performed using STATA9 software.

The prevalence of infection and 95% Bayesian credible intervals (BCI), adjusted for measurement error in serology, were also estimated in Batondo and Pabré using a Bayesian approach suggested by Joseph et al. (1995) [17]. We used two sets of priors for the sensitivity and specificity of the AgELISA test based on two studies conducted in infected pigs, one in Zambia and one in South Africa [6], [14].

### Ethical issues

The animal component of this protocol was approved by the Institutional Animal Care and Use Committee (IACUC) of the University of Oklahoma Health Sciences Center. No IACUC exists in Burkina Faso. The human component of this protocol was reviewed and approved by the ethical committee of the Center MURAZ (Ref. 02-2006/CE-CM) and by the Institutional Review Board of the University of Oklahoma Health Sciences Center (IRB# 12694) for both human and porcine participants. Informed consents for the interviews of participants and the provision of blood samples were obtained separately. The consent process was done orally because a very large proportion of the population had never been to school (62.5%). Oral consent was documented on the individual consent forms by the research staff (fingerprints were collected in place of signatures). Both IRBs approved the use of oral consents.

## Results

Socio-demographic characteristics of the three villages are described in Table 1. The majority of people in Batondo and Pabré drank water from traditional or open wells, whereas in Nyonyogo, most used water from bore wells. There were a few households in Batondo (1.2%) and Pabré (0.3%) in which people drank water from the river, marsh or spring. More than one-third of households in Pabré had latrines in contrast to Batonto and Nyonyogo where latrines were very rare. Wealth quintiles classified approximately one-half (50.9%) of households in Batondo among the poorest compared to around 1% in Pabré and Nyonyogo. In contrast, nearly 30% of households in Pabré and Nyonyogo were classified among the wealthiest compared to only 4.4% in Batondo.

**Table 1:** Households' socio-demographic characteristics and human seropositivity to *Taenia solium* antigens by village, Burkina Faso, 2007.

Variable	Category	Village		
		Batondo	Pabré	Nyonyogo
**Number of households sampled**		343	358	187
**Source of household drinking water**	Bore well/Tap water	102 (29.9)	149 (41.6)	177 (94.7)
	Cemented well	19 (5.6)	4 (1.1)	–
	Traditional well/open well	216 (63.3)	204 (57.0)	10 (5.3)
	River/Marsh/Spring	4 (1.2)	1 (0.3)	–
	Missing	2	0	0
**Presence of latrines in the household**	Yes	27 (7.8)	137 (38.4)	27 (14.4)
	Missing	0	1	0
**Result of the AgELISA test in one human selected at random in the household**	Positive	31 (10.3)	4 (1.4)	0 (0.0)
	Borderline	4 (1.3)	1 (0.3)	8 (4.5)
	Missing	43	71	11
**Wealth quintiles**	Mean ± std	−0.83±0.78	0.53±0.79	0.49±0.65
	1^st^ quintile (poorest)	(50.9)	(0.6)	(1.1)
	5^th^ quintile (wealthiest)	(4.4)	(29.8)	(29.0)
	Missing	3	1	1
**Presence of livestock**	Cattle	60 (17.4)	42 (11.7)	99 (52.9)
	Sheep	45 (13.0)	45 (12.6)	58 (31.0)
	Goats	195 (56.8)	187 (52.2)	153 (81.8)
	Donkey	31 (9.0)	236 (65.9)	152 (81.3)
	Poultry	260 (75.9)	247 (69.0)	139 (74.3)
	Pigs	228 (66.4)	189 (52.8)	5 (2.7)
	Missing	0	0	0

Characteristics related to pigs and pig management are reported in Table 2. Pigs were raised in two-thirds (66.4%) of the surveyed households in Batondo and in more than half (52.8%) of the surveyed households in Pabré. In Nyonyogo, most surveyed households owned ruminants and only 3% owned pigs. In Batondo, pigs were almost exclusively cared for by women (98.8%) whereas in Pabré, pigs were taken care of by men in nearly two-thirds of cases (63.1%). Similarly, the mother or eldest woman of the family was in charge of pigs in 71.7% of surveyed households in Batondo, but in only 25.0% of surveyed households in Pabré. Children were more often in charge of pigs in Pabré (10.5%) than in Batondo (1.8%). More than one-half of the surveyed households owning pigs in Pabré kept them penned during the rainy season. This was in contrast to Batondo where only 10.4% were kept penned, although over 90% were tethered. During the dry season, pigs were left to roam in all three villages to scavenge around cropped fields.

**Table 2:** Number(%) of pig management characteristics and pig serological results by village, Burkina Faso, 2007.

Variable	Category	Village		
		Batondo	Pabré	Nyonyogo
**Number of households raising pigs interviewed**		173	157	6
**Number of pigs per household**	Minimum number	1	1	1
	Median number	2	3	1
	Maximum number	24	18	7
	Mean ± std	3.2±3.0	3.6±2.7	3.0±2.8
	Missing	3	1	0
**Gender of the person in charge of pig management**	Female	171 (98.8)	56 (36.9)	-
	Male	2 (1.2)	96 (63.1)	6 (100)
	Missing	0	5	0
**Household role of the person in charge of pig management**	Father of the family	2 (1.2)	85 (55.9)	6 (100)
	Mother/eldest woman of the family	119 (71.7)	38 (25.0)	ND*
	One of the children	3 (1.8)	16 (10.5)	ND
	Other woman in the family	42 (25.3)	13 (8.6)	ND
	Missing	7	5	0
**Pigs are slaughtered at home (among household)**	Yes	8 (4.6)	108 (69.4)	2 (33.3)
	Missing	0	0	0
**Have you ever heard of white nodules that look like rice grains in pig meat?**	Yes	143 (82.7)	145 (92.9)	6 (100)
	Missing	0	1	0
**Purpose of pig raising**	To sell meat	1 (0.6)	7 (4.5)	(0.0)
	To sell live pigs	138 (79.8)	120 (76.9)	5 (83.3)
	Keep for reproduction	142 (82.1)	150 (96.1)	5 (83.3)
	Missing	0	1	0
**Pig management**	Keep in pen during rainy season	18 (10.4)	86 (55.1)	0 (0.0)
	Left roaming during rainy season	2 (1.2)	0 (0.0)	0 (0.0)
	Keep tethered during rainy season	163 (94.2)	74 (47.4)	6 (100)
	Keep in pen during dry season	1 (0.6)	15 (9.6)	0 (0.0)
	Left roaming during dry season	172 (99.4)	141 (90.4)	6 (100)
	Keep tethered during dry season	1 (0.6)	1 (0.6)	0 (0.0)
	Missing	0	1	0
**Result of the AgELISA for the detection of the larval stage of ** ***T. solium*** ** in pigs**	Borderline	3 (1.9)	5 (3.2)	0 (0.0)
	Positive	52 (32.5)	61 (39.6)	0 (0.0)
	Missing	13	3	1

A total of 336 pigs were sampled: 173 in Batondo, 157 in Pabré, and 6 in Nyonyogo. Four sera in Batondo, 3 in Pabré, and 1 in Nyonyogo were not tested because of insufficient volume. Using prior information on the sensitivity and specificity of the AgELISA from South Africa, the adjusted estimates of the seroprevalence of infection in Batondo and Pabré were 32.7% (95% BCI: 12.1%–54.3%) and 48.3% (95% BCI: 27.9%–74.8%), respectively. Using the priors from Zambia, the adjusted estimates of the seroprevalence of infection in Batondo and Pabré were 32.7% (95% BCI: 25.4%–68.3%) and 48.2% (95% BCI: 35.4%–82.6%), respectively. A total of 1.9% and 3.4% were found to be borderline positive in Batondo and Pabré, respectively. Neither serologically positive nor borderline animals were found in Nyonyogo.

The results of the multivariable logistic regression are reported in Table 3. Allowing pigs to roam during the rainy season was associated with an increased prevalence odds of infection when compared to keeping pigs penned (POR = 6.48, 95%CI: 1.23–34.17). Being aware of pig cysticercosis was also associated with an increased prevalence odds of infection (POR = 4.70, 95%CI: 1.76–12.52). The village, while not statistically significant, was retained in the model because it was a confounding factor for most of the other variables explored, including those retained in the final model. Once the model is adjusted for pig management during the rainy season and awareness of the infection, the prevalence odds of infection tended to be higher in Pabré than in Batondo (OR = 1.63, 95%CI: 0.94–2.83).

**Table 3:** Adjusted prevalence odds ratios (95% CIs) for pigs' seropositivity to *Taenia solium*, Burkina Faso, 2007.

Variable	Factor	Reference	POR	95% CI
**Pig rearing during the rainy season**	Tethered all the time	Penned all the time	1.47	0.80 – 2.69
	Roam some of the time	Penned all the time	6.48	1.23 – 34.17
**Has heard of porcine cysticercosis**	Yes	No	4.70	1.76–12.52
**Village**	Pabré	Batondo	1.63	0.94–2.83

POR: Prevalence odds ratio.

CI: Confidence interval.

## Discussion

This is the first community-based study conducted in Burkina Faso to report seroprevalence of porcine cysticercosis. Our results show that cysticercosis is highly prevalent in the two villages where pigs are commonly raised. These estimates are nearly 100 times higher than the previous estimate of 0.57% based on reports from meat inspection services [12]. This finding supports the opinion of Zoli *et al.* (2003) [10] that data from meat inspection are not representative of the real situation, mainly because the method is insensitive [14] and depends on the technical skills and the motivation of the examiner. Pigs infected with cysticercosis may also never be sold in the official market.

One-third (32.5%) of the pigs in Batondo and more than one-third (39.6%) in Pabré were infected, but none of the five sampled pigs in Nyonyogo was found to be positive. The latter is not surprising since pig breeding and pork consumption are very rare in this village. Similarly, Carabin et al. (2009) [13] reported very few cysticercosis seropositive inhabitants in Nyonyogo, and the few positives had all borderline reactions to the Ag ELISA.

Using priors from either South Africa or Zambia to adjust for measurement error resulted in the same median prevalence, but 95% BCI were shifted towards higher values when using the Zambia priors. This is because both the prior values for sensitivity and specificity from the Zambian study were higher than the South Africa values. The B158/B60 AgELISA test used here was recently shown to have the highest sensitivity and specificity for detection of cysticercosis-infected pigs when compared to tongue examination, EITB, and HP10 Ag-ELISA [6], [14]. The seroprevalences in Batondo and Pabré were comparable to that reported in pigs in Mozambique [5] but were higher than that reported in several other African countries [9], [15], [18], [19] and lower than the seroprevalence reported in pigs slaughtered on the clandestine market in Lusaka, Zambia [14] and in the Eastern Cape province in South Africa [6].

Pigs allowed to roam some of the time during the rainy season were more likely to be seropositive than pigs penned during all of the rainy season. This association was statistically significant even though the proportion of pigs allowed to roam during the rainy season was small because the association was so strong (POR = 6.48, 95% CI 1.23–34.17). Given the imprecision of this estimate, however, the association will need to be further explored. This observation agrees with the findings of Pondja *et al*. (2010) [5] who identified the free-range pig husbandry system as an important risk factor for porcine cysticercosis.

Conducting this pilot study lead to an appreciation that the pig management systems in Batondo and Pabré were different from what had been reported to us when the study was being planned. Indeed, in both of the two villages with pigs, animals were left roaming during dry seasons but restrained to one degree or another during crop production period (rainy season). The main difference in restraints was that pigs were confined in pens in Pabré and tethered to pillars in Batondo.

The difference in pig management, mainly during the rainy season, between Batondo and Pabré might reflect the difference in who owns and is responsible for raising the pigs. In Batondo, almost all pigs were owned by women whereas they were kept by men in two-third of the cases in Pabré. This result suggests that the target for intervention when planning future cysticercosis control strategies depends on who is responsible for pig raising in any given village.

Pigs owned by people who had heard of porcine cysticercosis were more likely to be seropositive than owners who were unaware of this infection. If pigs raised by the same farmers tend to become re-infected, this would suggest that people become aware of porcine cysticercosis when their pig is found to be infected.

Despite the high frequency of households with latrines in Pabré and the low prevalence of active infections in humans [13], the seroprevalence in pigs in this village tended to be higher than that in Batondo. This result is counter-intuitive but could be explained by the following. First, the presence of latrines does not certify their use because of foul odors, flies, flooding, etc. Second, latrines may be inappropriately built (closing door, septic tank) allowing access of pigs to human feces [20]. More research is needed to explain this observation.

It was anticipated that there would be an association of pig seropositivity and variables such as presence of a toilet in the household, household source of drinking water, and human serological status, but this was not confirmed by the statistical analysis. Indeed, the structure of community life in these villages means that any risk associated with lifestyle in a family is shared by other families nearby. For example, if a given family does not have access to latrines and thus defecates in nature, this will also affect the members of families equipped with latrines through environmental contamination. People with taeniasis may also infect a large number of pigs if they do not use latrines. Lescano *et al.* (2007) [21] demonstrated that a *T. solium* carrier can infect pigs within an area of 500 meters or more.

In conclusion, a high prevalence of porcine cysticercosis was observed in pig populations in Burkina Faso in villages with traditional pig-rearing practices. The co-occurrence of knowledge about cysticercosis and owning seropositive pigs suggests that information about this disease may not be available to livestock managers until the disease is already present. If so, this emphasizes the clear need for improving education in order to control this zoonosis.
